# Matrix Integral Approach to MIMO Mutual Information Statistics in High-SNR Regime †

**DOI:** 10.3390/e21111071

**Published:** 2019-11-01

**Authors:** Lu Wei, Chun-Hung Liu, Ying-Chang Liang, Zhidong Bai

**Affiliations:** 1 Department of Electrical and Computer Engineering, University of Michigan, Dearborn, MI 48128, USA; luwe@umich.edu; 2 Department of Electrical and Computer Engineering, Mississippi State University, Starkville, MS 39762, USA; chliu@ece.msstate.edu; 3 Center for Intelligent Networking and Communications, University of Electronic Science and Technology of China, Chengdu 611731, China; 4 MOEKLAS and School of Mathematics and Statistics, Northeast Normal University, Changchun 130024, China; baizd@nenu.edu.cn

**Keywords:** matrix integrals, MIMO mutual information, non-asymptotic analysis, random matrix theory

## Abstract

In this work, an analytical framework for deriving the exact moments of multiple-input- multiple-output (MIMO) mutual information in the high-signal-to-noise ratio (SNR) regime is proposed. The idea is to make efficient use of the matrix-variate densities of channel matrices instead of the eigenvalue densities as in the literature. The framework is applied to the study of the emerging models of MIMO Rayleigh product channels and Jacobi MIMO channels, which include several well-known channels models as special cases. The corresponding exact moments of any order for the high-SNR mutual information are derived. The explicit moment expressions are utilized to construct simple yet accurate approximations to the outage probability. Despite the high-SNR nature, simulation shows usefulness of the approximations with finite SNR values in the scenario of low outage probability relevant to MIMO communications.

## 1. Introduction

Mutual information is one of the most important quantities in information theory. It is crucial in the analysis and design of various communications and signal processing systems. In multiple-input-multiple-output (MIMO) communications, the supremum of the mutual information provides the fundamental performance measure of the channel capacity. Efforts have been made to understand the statistical properties of MIMO mutual information for different channel models. However, knowledge in the literature is essentially limited to either the exact mean values [1,2,3,4,5,6] or the limiting means and variances [7,8,9,10,11]. The first moment is relevant to the ergodic mutual information, whereas the higher order moments describe the outage probability essential to study to slow or block fading channels. Our study is also motivated by the fact that the prevailingly adopted asymptotic variances based approximative outage probabilities [8,9,10,11] fail to capture the true distribution when the number of antennas is small or the outage probability is low. Accurate characterizations require the exact higher order moments, which govern the tail of the distribution.

Deriving the exact higher order moments of MIMO mutual information for any given arbitrary signal-to-noise ratio (SNR) is a notable and longstanding challenging task. In fact, even the exact second moment is still unknown in the literature for any non-trivial MIMO channel model. The purpose of this paper is to show that, with the assumption of high-SNR, exact moments of any order for a wide class of MIMO channels can be explicitly obtained. The idea of the proposed approach stems from our observations that moment expressions of high-SNR mutual information can be efficiently obtained by means of integrals over matrix-valued channel densities. This is contrary to the existing approach [1,2,3,4,5,6,8,9,10,11], where the starting point is the seemingly simpler integrals over the eigenvalue densities of channel matrices.

To show the usefulness of the proposed framework, we study the mutual information of the MIMO Rayleigh product channels [3,4,5,7,10,12] and the Jacobi MIMO channels [6,9,11]. The MIMO Rayleigh product channel takes the well-known MIMO Rayleigh channels with [2] and without [1] correlation as special cases. The Jacobi MIMO channel is useful in modeling MIMO optical channels [6,11] and interference-limited multiuser channels [9]. The main results of this present work are the exact yet explicit formulas of all integer moments of mutual information for the above mentioned channel models in the high-SNR regime. We utilize the derived moments to construct analytical approximations to channel outage probabilities. Despite the high-SNR assumption, the resulting approximations turns out to be reasonably accurate for finite SNR values.

The high-SNR regime provides important insights into the statistical performance of MIMO channels. In particular, it characterizes the minimum required transmit power, which is also referred to as the high-SNR power offset [13]. The considered high-SNR mutual information is directly related to the high-SNR power offset, where its mean values have been derived for different channel models in [13]. As an application of our results, we may study as a possible future work the distribution of the power offset pertaining to the nonergodic channels, an open problem discussed in [13]. This open problem has been partially addressed in [14] for the case of a product of two MIMO Rayleigh channels.

## 2. Problem Statement

### 2.1. MIMO Mutual Information

Consider a MIMO system consisting of *n* transmit and *m* receive antennas, the channel in between is described by an m×n random matrix H. Assuming i.i.d. input across the transmit antennas and that the channel H is only known to the receiver, the mutual information in nats/second/Hz of the MIMO channel is [1]

(1) $$I=\ln\det\left(I_{m}+r{\mathrm{HH}}^{†}\right)=\sum_{i=1}^{m}\ln\left(1+r\theta_{i}\right),$$

where m≤n is assumed without loss of generality. In Equation (1), ln(·) is the natural logarithm, det(·) is the matrix determinant, *r* is the SNR, and $\theta_{m}\leq \cdots \leq \theta_{2}\leq \theta_{1}$ denote the eigenvalues of the Hermitian matrix ${\mathrm{HH}}^{†}$. In the high-SNR regime, by ignoring the constant $I_{m}$ in Equation (1) the mutual information is approximated by

(2) $$\begin{array}{ccc} I & = & m\ln r+\ln\det\left({\mathrm{HH}}^{†}\right) \end{array}$$

(3) $$\begin{array}{cc} = & m\ln r+\sum_{i=1}^{m}\ln\theta_{i}, \end{array}$$

where I denotes the approximation to I in the high SNR regime. This approximation becomes exact as the SNR *r* grows to infinity.

A fundamental information-theoretic quantity for MIMO channels is the outage probability, which is the probability that a given rate exceeds the value of the mutual information. For high-SNR mutual information Equation (2), the outage probability $P_{\mathrm{out}}\left(z\right)$ is defined as a function of the rate *z* as

(4) $$P_{\mathrm{out}}\left(z\right)=P\left(I<z\right).$$

Clearly, the above defined high-SNR outage probability $P_{\mathrm{out}}\left(z\right)$ is an approximation to the true outage probability $P\left(I<z\right)$, which approaches to its exact value as the SNR tends to infinity.

Finding simple and explicit expressions to the outage probability Equation (4) for finite-size systems of the following MIMO channel models is the focus of this work.

### 2.2. MIMO Rayleigh Product Channels

The MIMO Rayleigh product channel, originally proposed in [7], is a relevant model for the indoor wireless propagation in pico-cellular networks such as train stations, office buildings, and airports. Physical motivation for such a channel model can be found, for example, in ([15], Section 3). The MIMO Rayleigh product channel has received increasing attention due to the recent breakthrough in understanding its finite-size distribution [3,4,5]. Assuming a MIMO channel with $d_{0}$ transmit and $d_{M}$ receive antennas, the information transmitted to the receiver goes through M−1 successive layers, each having $d_{i}$ (i=1,⋯,M−1) scatterers, the corresponding channel equals the product of *M* channel matrices

(5) $$H=H_{M}\cdots H_{1}.$$

The dimensions of the *i*-th channel $H_{i}$ is $d_{i}\times d_{i-1}$, where each channel is assumed to be an independent MIMO Rayleigh channel. All the scattering between the MIMO Rayleigh channels $H_{i}$ and $H_{i+1}$ happens through the $d_{i}$ scatterers in the layer *i*, which can be thought as $d_{i}$ keyholes. In the literature, the channel model considered in Equation (5) has been referred to different names such as multiple cluster scattering channel, progressive scattering channel, or multiple Rayleigh scattering channel. We choose to use the term MIMO Rayleigh product channel in this paper to emphasize that each channel in the product is described by the Rayleigh fading.

The effect of spatial correlation among receive antennas is considered, which is caused by physical constraints of the terminal size. We choose the separable correlation model, where the effect of correlation is multiplicative, i.e.,

(6) $$E\left[HH^{†}\right]=\left(\prod_{i=1}^{M}d_{i-1}\right)\Sigma ,$$

where the $d_{M}\times d_{M}$ deterministic matrix Σ is the correlation matrix. For convenience of the discussion, we study here the receiver side correlation, whereas the transmitter side correlation can be considered at the same time. The only difference is that the result (21a) will have an additional term lndetΩ, where the $d_{0}\times d_{0}$ matrix Ω specifies the correlation matrix at the transmitter side. As will be discussed in detail in Section 3, one could assume without loss of generality that the dimensions of the channels are ordered as

(7) $$\nu_{i}=d_{i}-d_{M}\geq 0,\;\;\;\;i=0,\cdots ,M-1,$$

which is known as the weak commutation relation for products of random matrices [4]. Strictly speaking, the weak commutation relation was established in [4] for the case $\Sigma =I_{m}$. The extension to an arbitrary Σ can be seen by the fact that the parametrization ([4], Equations (2)–(4)) is essentially the same in the presence of Σ. The extension is further confirmed by the observation that the density (9) is indeed invariant under any permutation of the parameters $\nu_{i}$. For notational convenience, we further denote the smallest dimension $d_{M}$ by

(8) $$m=d_{M}.$$

By the above assumptions, the density of the joint eigenvalues of the hermitian matrix ${\mathrm{HH}}^{†}$ is given by [5]

(9) $$p\left(\theta \right)=\frac{1}{c}\det\left(\theta_{i}^{j-1}\right)\det\left(f_{j}\left(\theta_{i}\right)\right),\;\;\;\;i,j=1,\cdots ,m,$$

with

(10) $$f_{j}\left(\theta \right)=G_{0,M}^{M,0}\left(\frac{\theta }{\sigma_{j}}\left|\begin{array}{c} -\\ \nu_{M-1},\ldots ,\nu_{0} \end{array}\right.\right),$$

where $0\leq \theta_{m}\leq \cdots \leq \theta_{2}\leq \theta_{1}<\infty$ and *c* is a normalization constant that does not depend on θ. Here, $\sigma_{j}$ denotes the *j*-th eigenvalue of the correlation matrix Σ with $\sigma_{i}\neq \sigma_{j}$ for i≠j. The case when some of $\sigma_{i}$ are equal can be resolved by the L’Hopital’s rule. The function in Equation (10) is a Meijer’s G-function [5] and the determinant in Equation (9)

(11) $$\det\left(\theta_{i}^{j-1}\right)=\prod_{1\leq i<j\leq m}\left(\theta_{i}-\theta_{j}\right)$$

is a Vandermonde determinant.

The MIMO Rayleigh product channel, seen in Equations (5) and (6), is a general channel model that includes the following well-known models as special cases:

- Uncorrelated MIMO Rayleigh product channels [3,4,7,10] when $\Sigma =I_{m}$,
- MIMO Rayleigh channels [2] when M=1,
- Uncorrelated MIMO Rayleigh channels [1,8,9] when M=1 and $\Sigma =I_{m}$.

In the literature, the exact ergodic mutual information $E\left[I\right]$ of the MIMO Rayleigh product channels with and without correlation has been derived in [3,4,5], respectively. For the MIMO Rayleigh channels, the exact ergodic mutual information with and without correlation has been derived in [1,2], respectively. For the exact higher order moments $E\left[I^{k}\right]$, k=2,3,⋯, that are needed to characterize the outage probability, no explicit finite-size results seem available for any of the above channel models. We exclude the discussion of finite-size results in the literature that involve, as final results, integral representations or combinatorial objects such as determinants and sums over partitions. For example, for different channel models in [16,17] the exact outage mutual information has been represented as contour integrals involving determinants, which may only be evaluated numerically in most cases. The existing results are limited to a scenario of large channel dimensions, where some asymptotic formulas for the second moment are available [8,9,10].

### 2.3. Jacobi MIMO Channels

The Jacobi MIMO channel is a useful model for the MIMO optical communications [6,11] as well as the interference-limited multiuser MIMO [9]. We will, however, formulate the problem mainly in the context of the former application, where the relevance to the latter will be briefly discussed.

The spatial degrees of freedom of the MIMO Rayleigh channels (and its generalization to MIMO product channels) lead to the well-known capacity scaling law [1]. The idea behind the MIMO fiber optical channels is to attain a similar scaling law by exploiting the spatial degrees of freedom as well. Particularly, multiple spatial transmission in the same fiber is possible by designing a multi-mode and/or multi-core fiber. To explore the spatial diversity, the Jacobi MIMO optical channel was proposed in [6,11], which relies on the following assumptions. The propagation through the fiber is assumed to be as lossless such that it can be modeled as an l×l random unitary matrix ${\mathrm{UU}}^{†}=I_{l}$, also known as the scattering matrix. Assuming *n* transmitting and *m* receiving modes (with m≤n), the effective optical MIMO channel is given by the upper left sub-matrix of the scattering matrix $U=\left(u_{ij}\right)$ with the condition that l≥m+n, i.e.,

(12) $$H={\left(u_{ij}\right)}_{i=1,\cdots ,m;\;j=1,\cdots ,n}.$$

With these assumptions, the joint density of the eigenvalues of the hermitian channel matrix ${\mathrm{HH}}^{†}$ is expressed as [6,11]

(13) $$p\left(\theta \right)=\frac{1}{c}\prod_{1\leq i<j\leq m}{\left(\theta_{i}-\theta_{j}\right)}^{2}\prod_{i=1}^{m}\theta_{i}^{\alpha_{1}}{\left(1-\theta_{i}\right)}^{\alpha_{2}},$$

where $0\leq \theta_{m}\leq \cdots \leq \theta_{2}\leq \theta_{1}\leq 1$,

(14) $$\alpha_{1}=n-m,\;\;\;\;\alpha_{2}=l-m-n,$$

and *c* is a normalization constant that does not depend on θ. The ensemble Equation (13) is referred to as the Jacobi ensemble [18] in random matrix theory, and hence the name Jacobi MIMO channels in the information theory and communications theory communities. Notice that when the SNR is high enough the optical channel becomes nonlinear which may not be modeled by the above channel model. Fortunately, our derived analytical results are valid for moderate high SNR values as shown in Section 4.

The eigenvalue density of the interference-limited MIMO channel considered in [9] takes the form of Equation (13) with the same parameter $\alpha_{1}=n-m$ as the difference between the number of transmit and receive antennas. For this application, the parameter $\alpha_{2}$ is now

(15) $$\alpha_{2}=kn-m,$$

where *k* denotes the number of interferers. For a detailed discussion of the interference-limited MIMO channels and its connection to the Jacobi ensemble, we refer to [9].

For applications in optical MIMO communications, the exact ergodic mutual information $E\left[I\right]$ of the Jacobi MIMO channels was derived in [6], whereas an unexplicit as well as a limiting second moment formulas can be found in [11]. Note that the exact higher order moments might be also derived from the representation in ([11], Equation (13)), which would carry the same combinatorial structure involving sums over partitions. On the contrary, our proposed moments expressions for high SNRs in Proposition 2 are simple and explicit, the difficulty of which does not increase with the order of moment considered. For applications to interference-limited MIMO channels, the first two limiting moments and a differential equation for the moments were obtained in [9]. Finally, we note that the random variable $\ln\det\left({\mathrm{HH}}^{†}\right)$ for Jacobi MIMO channels was shown in ([19], Proposition 2.4) to have the same distribution as a product of independent Beta random variables, whose density function can be obtained in principle, albeit complicated, by multiple convolutions of the underlying Beta densities.

## 3. Exact Moments of High-SNR Mutual Information

Despite that even the exact second moment $E\left[I^{2}\right]$ of the mutual information Equation (1) is difficult to obtain for any previously discussed channel model, we will show that the exact moments $E\left[I^{k}\right]$, k=1,2,⋯, of the high-SNR mutual information Equation (2) can be explicitly calculated. These moments will be utilized to construct simple and accurate approximations to the outage probability of the considered MIMO channels.

To compute the moments, one naturally starts from integrals involving the eigenvalue densities, seen in Equations (9) and (13) as has been done in the literature [1,2,3,4,5,6,8,9,10,11]. Contrary to this prevailing approach, our starting point is the integrals over the matrix-variate densities of the channel matrices ${\mathrm{HH}}^{†}$. This may be counter-intuitive as the matrix integral involves to the order of $m^{2}$ real variables, whereas the integral over eigenvalue density only involves *m* variables. We will show, however, that by starting from matrix integrals the exact moment of any order can be derived in a straightforward manner.

As mlnr in Equation (2) is a constant, we first focus on the random variable

(16) $$X=\ln\det\left({\mathrm{HH}}^{†}\right).$$

The cumulant generating function K(s) of *X* is defined as

(17) $$K\left(s\right)=\ln E\left[e^{sX}\right]=\sum_{i=1}^{\infty }{\tilde{\kappa }}_{i}\frac{s^{i}}{i!},$$

where the *i*-th order cumulant ${\tilde{\kappa }}_{i}$ of *X* can be recovered from the generating function as

(18) $${\tilde{\kappa }}_{i}=\frac{\;d^{i}}{\;ds^{i}}K\left(s\right){|}_{s=0}.$$

Denote the *i*-th order cumulant of I by $\kappa_{i}$, we have

(19a) $$\begin{array}{ccc} \kappa_{1} & = & m\ln r+{\tilde{\kappa }}_{1}, \end{array}$$

(19b) $$\begin{array}{ccc} \kappa_{i} & = & {\tilde{\kappa }}_{i},\;\;\;\;i\geq 2, \end{array}$$

which is due to the shift-equivariant and the shift-invariant property, respectively. With the knowledge of cumulants, the corresponding moments can be determined. In general, the *i*-th order moment is an *i*-th degree polynomial in the first *i* cumulants and vice versa. For example, the first five moments of I written in terms of its first five cumulants are listed below

(20a) $$\begin{array}{ccc} E\left[I\right] & = & \kappa_{1}, \end{array}$$

(20b) $$\begin{array}{ccc} E\left[I^{2}\right] & = & \kappa_{2}+\kappa_{1}^{2}, \end{array}$$

(20c) $$\begin{array}{ccc} E\left[I^{3}\right] & = & \kappa_{3}+3\kappa_{2}\kappa_{1}+\kappa_{1}^{3}, \end{array}$$

(20d) $$\begin{array}{ccc} E\left[I^{4}\right] & = & \kappa_{4}+4\kappa_{3}\kappa_{1}+3\kappa_{2}^{2}+6\kappa_{2}\kappa_{1}^{2}+\kappa_{1}^{4}, \end{array}$$

(20e) $$\begin{array}{ccc} E\left[I^{5}\right] & = & \kappa_{5}+5\kappa_{4}\kappa_{1}+10\kappa_{3}\kappa_{2}+10\kappa_{3}\kappa_{1}^{2}+15\kappa_{2}^{2}\kappa_{1}+10\kappa_{2}\kappa_{1}^{3}+\kappa_{1}^{5}. \end{array}$$

We now present the main technical contributions of this paper, which are summarized in the two propositions and two corollaries below.

### 3.1. All the Integer Moments of Mutual Information of MIMO Rayleigh Product Channels

**Proposition** **1.** *The i-th exact cumulant $\kappa_{i}$ of the high-SNR mutual information (Equation (2)) of the MIMO Rayleigh product channels (Equation (5)) is given by*

(21a) $$\begin{array}{cc} \kappa_{1}= & m\sum_{j=1}^{M}\psi_{0}\left(d_{j-1}-m\right)+\sum_{j=1}^{M}d_{j-1}\left(\psi_{0}\left(d_{j-1}\right)-\psi_{0}\left(d_{j-1}-m\right)\right)+\\ & m(\ln r-M)+\ln\det\Sigma , \end{array}$$

(21b) $$\begin{array}{cc} \kappa_{i}= & m\sum_{j=1}^{M}\psi_{i-1}\left(d_{j-1}-m\right)+\sum_{j=1}^{M}d_{j-1}\left(\psi_{i-1}\left(d_{j-1}\right)-\psi_{i-1}\left(d_{j-1}-m\right)\right)+\\ & \left(i-1\right)\sum_{j=1}^{M}\left(\psi_{i-2}\left(d_{j-1}\right)-\psi_{i-2}\left(d_{j-1}-m\right)\right),\;\;\;\;i\geq 2, \end{array}$$

*where*

(22) $$\psi_{i}\left(z\right)=\frac{\partial^{i+1}\ln\Gamma \left(z\right)}{\partial z^{i+1}}={\left(-1\right)}^{i+1}i!\sum_{k=0}^{\infty }\frac{1}{{\left(k+z\right)}^{i+1}}$$

*denotes the i-th order polygamma function [20].*

The proof of Proposition 1 is in the Appendix A. Note that for positive integer arguments, the polygamma function of order 0 (digamma function) reduces to a finite sum as

(23) $$\psi_{0}\left(l\right)=-\gamma +\sum_{k=1}^{l-1}\frac{1}{k}$$

with γ≈0.5772 being the Euler’s constant, and the polygamma functions of order i≥1 in Equation (24) also become finite sums as

(24) $$\psi_{i}\left(l\right)={\left(-1\right)}^{i+1}i!\left(\zeta \left(i+1\right)-\sum_{k=1}^{l-1}\frac{1}{k^{i+1}}\right),$$

where

(25) $$\zeta \left(s\right)=\sum_{k=1}^{\infty }\frac{1}{k^{s}}$$

is the Riemann zeta function. In particular, the trigamma function (i=1) reduces to

(26) $$\psi_{1}\left(l\right)=\frac{\pi^{2}}{6}-\sum_{k=1}^{l-1}\frac{1}{k^{2}}.$$

Note also that an exact moment expression of the MIMO Rayleigh product channel has been recently obtained in ([12], Proposition 4), which involves a combinatorial structure of matrix determinant. On the contrary, our proposed moment expressions are simple and explicit partially due to the efficient use of the cumulant generating function, an approach we try to advocate in this paper.

The general results in Proposition 1 can be simplified to various special cases depending on the channel models considered. The following two special cases, as summarized in the Corollaries 1 and 2, may deserve separate attention.

**Corollary** **1.** *In the case of square channel matrices*

(27) $$d_{0}=d_{1}=\cdots =d_{M}=m,$$

*the results of Proposition 1 are simplified to*

(28a) $$\begin{array}{ccc} \kappa_{1} & = & M\left(m\psi_{0}\left(m\right)-m+1\right)+m\ln r+\ln\det\Sigma , \end{array}$$

(28b) $$\begin{array}{ccc} \kappa_{i} & = & M\left(m\psi_{i-1}\left(m\right)+\left(i-1\right)\left(\psi_{i-2}\left(m\right)-\psi_{i-2}\left(1\right)\right)\right),\;\;\;\;i\geq 2. \end{array}$$

The proof of Corollary 1 is in Appendix B. Note that the above channel model corresponds to the scenario of equal number of scatterers on each layer (cf. Equation (5)). Note also that Proposition 1 is in fact not directly valid in the case of Equation (27), where some general expressions from Equations (A22a) and (A23a) will be needed, cf. Equations (A4) and (A5).

**Corollary** **2.** *In the case M=1, i.e., the MIMO Rayleigh channels, the results of Proposition 1 reduce to*

(29a) $$\begin{array}{ccc} \kappa_{1} & = & \left(m-d_{0}\right)\psi_{0}\left(d_{0}-m\right)+d_{0}\psi_{0}\left(d_{0}\right)+m\left(\ln r-1\right)+\ln\det\Sigma , \end{array}$$

(29b) $$\begin{array}{ccc} \kappa_{i} & = & \left(m-d_{0}\right)\psi_{i-1}\left(d_{0}-m\right)+d_{0}\psi_{i-1}\left(d_{0}\right)+\left(i-1\right)\left(\psi_{i-2}\left(d_{0}\right)-\psi_{i-2}\left(d_{0}-m\right)\right),\;i\geq 2. \end{array}$$

The proof of Corollary 2 follows directly by setting M=1 in Equation (21). In the literature, the first two (unsimplified) cumulants $\kappa_{1}$ and $\kappa_{2}$ of Corollary 2 have been reported in [13,21,22], where some bounds on the corresponding outage probability can be also found in [22]. In particular, the variance formulas provided in ([22], Section IV) can be further simplified to finite sums. Note that the derivation in [22] essentially ended with the expressions for the moment generating functions, whereas we have derived explicit expressions for all the moments via the corresponding cumulants. In addition, in our construction of the outage probability from the moments no generic probabilistic bounds, such as the Chernoff bound used in [22], are needed.

It is interesting to observe that the terms involving SNR γ and spatial correlation Σ only appear in the first cumulants in Equations (21a), (28a) and (29a) of the high-SNR mutual information. As a result, the corresponding cumulants for the uncorrelated channel models only incur a change in Equations (21a), (28a) and (29a) by setting

(30) $$\ln\det\Sigma =\ln\det I_{m}=0,$$

whereas the higher cumulants in Equations (21b), (28b) and (29b) remain the same.

### 3.2. All the Integer Moments of Mutual Information of Jacobi MIMO Channels

**Proposition** **2.** *The i-th exact cumulant $\kappa_{i}$ of the high-SNR mutual information in Equation (2) of the Jacobi MIMO channels in Equation (12) is given by*

(31a) $$\begin{array}{ccc} \kappa_{1} & = & n\psi_{0}\left(n\right)-l\psi_{0}\left(l\right)-\left(n-m\right)\psi_{0}\left(n-m\right)+\left(l-m\right)\psi_{0}\left(l-m\right)+m\ln r, \end{array}$$

(31b) $$\begin{array}{ccc} \kappa_{i} & = & n\psi_{i-1}\left(n\right)-l\psi_{i-1}\left(l\right)-\left(n-m\right)\psi_{i-1}\left(n-m\right)+\left(l-m\right)\psi_{i-1}\left(l-m\right)+\\ & & \left(i-1\right)\left(\psi_{i-2}\left(n\right)-\psi_{i-2}\left(l\right)-\psi_{i-2}\left(n-m\right)+\psi_{i-2}\left(l-m\right)\right),\;\;\;\;i\geq 2. \end{array}$$

The proof of Proposition 2 can be found in Appendix C. A similar, but unsimplified, $\kappa_{1}$ expression was obtained in the context of mean power offset of interference-limited MIMO channels ([13], Equation (78)). In such a setting, our derived higher cumulants may useful in the study of the distribution of power offset, which may be addressed separately as a future work.

## 4. Moment-Based Approximations to Outage Probability

With the derived exact cumulant expressions from Equations (21), (28), (29), (31) and the cumulant-moment relations Equation (20), moment-based approximations to the outage probability in Equation (4) for each of the discussed channel model can now be constructed. The basic idea of moment based approximation is to match the moments and support of an intractable distribution by an elementary distribution and the associated orthogonal polynomials [23,24]. The theory of moment-based approximation as developed by Ha and Provost [23,24] assigns Gaussian, Gamma, or Beta density as the elementary distribution (initial approximation) when the considered random variable is supported in (−∞,∞), [a,∞), or [a,b] (*a*, *b* being finite), respectively. The respective orthogonal polynomials for the chosen elementary densities are Hermite, Laguerre, and Jacobi polynomials. The parameters of the initial approximations are obtained by matching the first two moments, whereas the orthogonal polynomials encode the higher moments. An important property is that the approximation accuracy in general improves as the number of moments involved increases. Note also that the moment-based approximation provides closed-form distribution functions by taking the moment expressions as input, where no numerical simulation of any random variable is needed.

Since the random variable of interest in Equation (16) is supported in X∈[a,∞) for the MIMO Rayleigh product channels and in X∈(−∞,0] for the Jacobi MIMO channels, the gamma distribution is chosen as the elementary distribution of the approximation. As a consequence, the Laguerre polynomials as the associated orthogonal polynomials are utilized to construct the approximation. The resulting approximative distribution function $F_{q}\left(x\right)$ to the distribution function P(X<x) of the random variable *X* in Equation (16), obtained by matching the first *q* moments of *X* to a linear combination of Laguerre polynomials up to degree *q*, can be read off from ([23], Appendix 2) as

(32) $$F_{q}\left(x\right)=\frac{\gamma \left(\alpha ,x/\beta \right)}{\Gamma (\alpha )}+\epsilon_{q}\left(x\right),$$

where

(33) $$\epsilon_{q}\left(x\right)=\sum_{i=3}^{q}w_{i}\sum_{j=0}^{i}\frac{{\left(-1\right)}^{j}\Gamma \left(\alpha +i\right)}{(i-j)!j!}\frac{\gamma \left(\alpha +j,x/\beta \right)}{\Gamma (\alpha +j)}$$

with

(34) $$w_{i}=\sum_{l=0}^{i}\frac{{\left(-1\right)}^{l}i!}{\left(i-l\right)!l!\Gamma \left(\alpha +l\right)\beta^{l}}E\left[X^{l}\right]$$

and

(35) $$\gamma \left(a,b\right)=\int_{0}^{b}t^{a-1}e^{-t}\;dt$$

being the lower incomplete Gamma function. The parameters α, β in the approximative distribution function in Equation (32) are found by matching the first two moments of *X* to a Gamma random variable having a density

(36) $$\frac{1}{\Gamma \left(\alpha \right)\beta^{\alpha }}x^{\alpha -1}e^{-\frac{x}{\beta }},\;\;\;\;x\in \left[0,\infty \right)$$

as

(37a) $$\begin{array}{ccc} \alpha & = & \frac{E^{2}\left[X\right]}{E\left[X^{2}\right]-E^{2}\left[X\right]}=\frac{{\left(\kappa_{1}-m\ln r\right)}^{2}}{\kappa_{2}}, \end{array}$$

(37b) $$\begin{array}{ccc} \beta & = & \frac{E\left[X^{2}\right]-E^{2}\left[X\right]}{E\left[X\right]}=\frac{\kappa_{2}}{\kappa_{1}-m\ln r}, \end{array}$$

where the derived higher cumulant expressions enter the approximation via Equation (34), cf. Equation (20). Finally, the resulting approximation to the outage probability of Equation (4) is obtained by keeping in mind the relation of Equation (2) as

(38) $$P_{\mathrm{out}}\left(z\right)\approx F_{q}\left(z-m\ln r\right).$$

The simplest form of the moment-based approximation corresponds to q=2 in Equation (32) as

(39) $$F_{2}\left(x\right)=\frac{\gamma \left(\alpha ,x/\beta \right)}{\Gamma (\alpha )},$$

where only the first two moments are involved. Note that the above Equations (32)–(39) are valid for X∈[0,∞) of the MIMO Rayleigh product channels and its special cases. For the Jacobi MIMO channels having X∈(−∞,0], one constructs an approximation to the random variable −X in a parallel manner as the above with details omitted.

In principle, with the number of moments $E\left[X^{i}\right]$, i=1,⋯, involved in the term in Equation (34) increases, the accuracy of the approximation in Equation (38) improves [23,24]. This is especially true for the tail of the distribution, where the accuracy is governed by the higher order moments. We now focus on the numerical study of the approximation accuracy in various realistic scenarios. The emphasis will be on the low outage probability in the regime when the SNR and the number of antennas are finite. We point out that the outage probability at low outage is a crucial metric for optical communications, where requesting a retransmission in the case of packet loss may not be possible [11].

**Figure 1:**
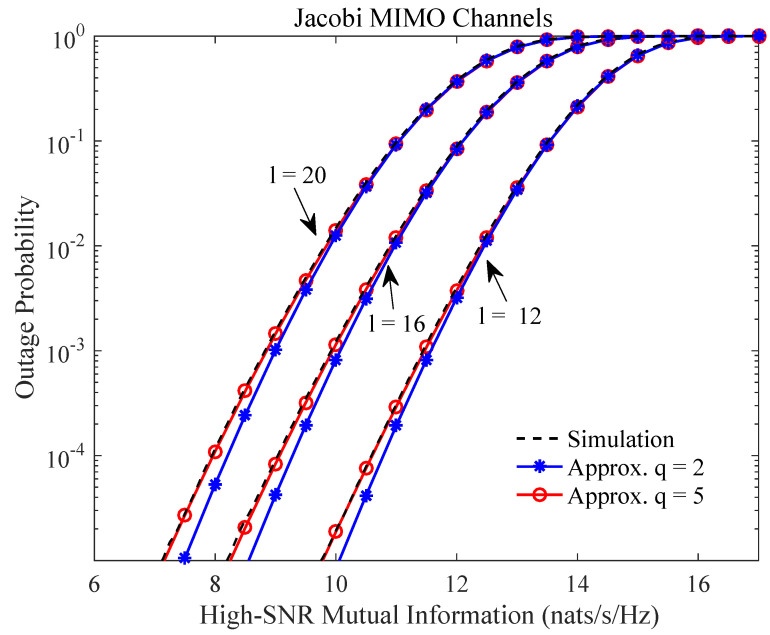
Outage probability of high-signal-to-noise ratio (SNR) mutual information (Equation (2)) of Jacobi multiple-input-multiple-output (MIMO) channels (Equation (12)) for different *l* with m=4, n=6, and r=20 dB.

We first study the impact of the number of moments *q* on the approximation accuracy, where we consider the Jacobi MIMO channels Equation (12) for illustration. Figure 1 shows the outage probability of the high-SNR mutual information Equation (2) assuming the channel dimensions m=4 and n=6, where different dimensions l=12, 16, and 20 of the scattering matrices are considered. The numerical simulations are compared with the moment-based approximative outage probability, where the number of moments considered are q=2 and q=5. It is observed that the accuracy of the proposed approximation improves (most prominently in the tails) as the number of moments increases from q=2 to q=5. We also see that the outage probability decreases as the number of untapped channels l−m−n decreases, where the same phenomenon has been observed in [11] as well.

**Figure 2:**
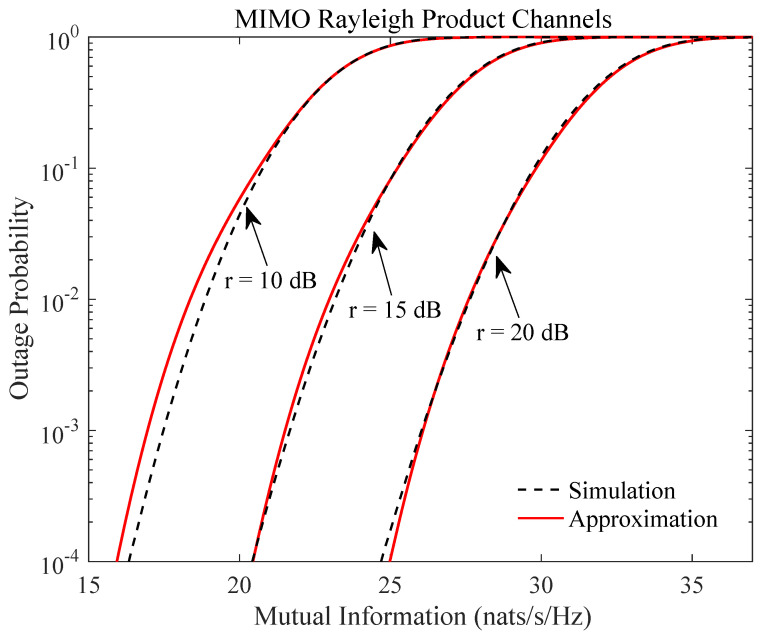
Outage probability of mutual information Equation (1) of MIMO Rayleigh product channels Equation (5) for different SNR values with q=5, M=3, $d_{0}=7$, $d_{1}=6$, $d_{2}=5$, $d_{3}=4$, and ρ=0.7.

**Figure 3:**
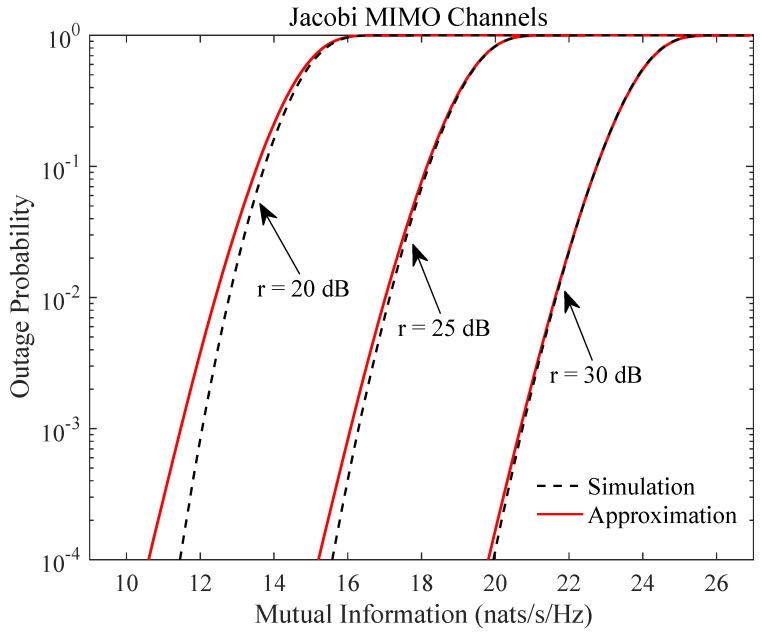
Outage probability of mutual information Equation (1) of Jacobi MIMO channels Equation (12) for different SNR values with q=5, l=12, m=4, and n=6.

We now study the impact of finite SNR on the accuracy of the outage probability. Since the approximation is formally valid when the SNR approaches infinity, the study helps evaluate the usefulness of the results in practical scenarios of finite SNR values. In Figure 2, we consider the MIMO Rayleigh product channels in Equation (5) with M=3, $d_{0}=7$, $d_{1}=6$, $d_{2}=5$, $d_{3}=4$, where the exponential correlation model

(40) $${\left(\Sigma \right)}_{i,j}=\rho^{\left|i-j\right|},\;\;\;\;\rho \in \left[0,1\right),$$

is chosen for the correlation matrix Σ with ρ being set at 0.7. In Figure 3, we consider the Jacobi MIMO channels in Equation (5) with the channel dimensions l=12, m=4, and n=6. In both figures, the number of moments used in the approximation equals q=5. Despite the high-SNR assumption, it is observed that the proposed approximation is already reasonably accurate for not-so-high SNR values even for outage probability as low as $10^{-4}$. Simulations with other parameter values have been extensively performed, where similar accuracy performance as in Figure 2 and Figure 3 persists.

## 5. Conclusions

In this work, we study the mutual information of MIMO Rayleigh product channels and Jacobi MIMO channels, which has received substantial attention very recently. For the considered channel models, the exact moments of any order of the high-SNR mutual information are derived. The results are derived by making use of the relevant matrix-variate densities of the channel matrices. The obtained exact moments lead to closed-form approximations to the outage probability. Simulations demonstrate the usefulness of the results in practical scenarios of low outage probability with finite SNR values and channel dimensions. Future work includes extending the analytical framework to studying the moments of mutual information for MIMO channels with more complicated matrix-variate densities such as the MIMO Rician channels.

## Appendix A. Proof of Proposition 1

The proofs of Propositions 1 and 2 as well as Corollary 1 rely on the matrix-variate gamma and beta distributions as well as some finite sum identities involving polygamma functions, which are summarized in the definitions and the lemma below.

**Definition** **A1.**
*The matrix-variate gamma density with parameters (α,Σ) is defined as ([18], p. 357)*

(A1) $$\frac{1}{\Gamma_{m}\left(\alpha \right)\det{\left(\Sigma \right)}^{\alpha }}\det({X)}^{\alpha -m}e^{-\mathrm{tr}\left(\Sigma^{-1}X\right)},$$

*where X and Σ are Hermitian matrices of dimensions m×m, and*

(A2) $$\Gamma_{m}\left(\alpha \right)=\pi^{\frac{1}{2}m\left(m-1\right)}\prod_{k=0}^{m-1}\Gamma \left(\alpha -k\right)$$

*denotes the multivariate gamma function ([18], p. 356).*

**Definition** **A2.**
*The matrix-variate beta density with parameters (α,β) is defined as ([18], p. 357)*

(A3) $$\frac{\Gamma_{m}\left(\alpha +\beta \right)}{\Gamma_{m}\left(\alpha \right)\Gamma_{m}\left(\beta \right)}\det{\left(X\right)}^{\alpha -m}\det{\left(I_{m}-X\right)}^{\beta -m},$$

*where X is a Hermitian matrix of dimensions m×m.*

**Lemma** **A1.**
*For an integer l>0, we have*

(A4a) $$\begin{array}{ccc} \sum_{k=1}^{n}\psi_{0}\left(k+l\right) & = & \left(n+l\right)\psi_{0}\left(n+l\right)-l\psi_{0}\left(l\right)-n, \end{array}$$

(A4b) $$\begin{array}{ccc} \sum_{k=1}^{n}\psi_{i}\left(k+l\right) & = & \left(n+l\right)\psi_{i}\left(n+l\right)-l\psi_{i}\left(l\right)+i\left(\psi_{i-1}\left(n+l\right)-\psi_{i-1}\left(l\right)\right),\;\;\;i\geq 1, \end{array}$$

*and when l=0, we have*

(A5a) $$\begin{array}{ccc} \sum_{k=1}^{n}\psi_{0}\left(k\right) & = & n\psi_{0}\left(n\right)-n+1, \end{array}$$

(A5b) $$\begin{array}{ccc} \sum_{k=1}^{n}\psi_{i}\left(k\right) & = & n\psi_{i}\left(n\right)+i\left(\psi_{i-1}\left(n\right)-\psi_{i-1}\left(1\right)\right),\;\;\;\;i\geq 1. \end{array}$$

**Proof.** We will first prove the identities of Equation (A4), whereas the identities of Equation (A5) can then be established by taking appropriate limits of Equation (A4) to resolve the indeterminacy. To prove Equation (A4a), we observe from the definition of digamma function of Equation (23) that

(A6) $$\psi_{0}\left(k+l\right)=\psi_{0}\left(l\right)+\sum_{i=0}^{k-1}\frac{1}{l+i},$$

which gives

(A7a) $$\begin{array}{ccc} \sum_{k=1}^{n}\psi_{0}\left(k+l\right) & = & n\psi_{0}\left(l\right)+\sum_{k=1}^{n}\sum_{i=0}^{k-1}\frac{1}{l+i} \end{array}$$

(A7b) $$\begin{array}{ccc} & \;\;\;\;\;= & n\psi_{0}\left(l\right)+\sum_{i=0}^{n-1}\sum_{k=i+1}^{n}\frac{1}{l+i} \end{array}$$

(A7c) $$\begin{array}{ccc} & \;\;\;\;\;\;\;\;= & n\psi_{0}\left(l\right)+n\sum_{i=0}^{n-1}\frac{1}{l+i}-\sum_{i=1}^{n-1}\frac{i}{l+i} \end{array}$$

(A7d) $$\begin{array}{ccc} & \;\;\;\;\;\;\;\;\;\;\;\;\;= & n\psi_{0}\left(l\right)+n\left(\psi_{0}\left(n+l\right)-\psi_{0}\left(l\right)\right)-\sum_{i=1}^{n-1}\frac{l+i-l}{l+i} \end{array}$$

(A7e) $$\begin{array}{ccc} & \;\;\;\;\;\;\;\;= & n\psi_{0}\left(n+l\right)+l\sum_{i=1}^{n-1}\frac{1}{l+i}-n+1 \end{array}$$

(A7f) $$\begin{array}{ccc} & \;\;\;\;\;\;\;\;\;\;\;\;= & n\psi_{0}\left(n+l\right)+l\left(\psi_{0}\left(n+l\right)-\psi_{0}\left(l+1\right)\right)-n+1 \end{array}$$

(A7g) $$\begin{array}{ccc} & \;\;\;\;\;\;\;= & \left(n+l\right)\psi_{0}\left(n+l\right)-l\psi_{0}\left(l\right)-n. \end{array}$$

This proves Equation (A4a). To show Equation (A4b), by the series representation of polygamma function of Equation (24), one has

(A8) $$\psi_{i}\left(k+l\right)=\psi_{i}\left(l\right)+{\left(-1\right)}^{i}i!\sum_{j=0}^{k-1}\frac{1}{{\left(l+j\right)}^{i+1}},$$

which similarly gives

(A9a) $$\begin{array}{ccc} \sum_{k=1}^{n}\psi_{i}\left(k+l\right) & = & n\psi_{i}\left(l\right)+{\left(-1\right)}^{i}i!\sum_{j=0}^{n-1}\sum_{k=j+1}^{n}\frac{1}{{\left(l+j\right)}^{i+1}} \end{array}$$

(A9b) $$\begin{array}{ccc} & \;\;\;\;\;\;\;\;\;\;\;\;= & n\psi_{i}\left(l\right)+{\left(-1\right)}^{i}i!n\sum_{j=0}^{n-1}\frac{1}{{\left(l+j\right)}^{i+1}}-{\left(-1\right)}^{i}i!\sum_{j=1}^{n-1}\frac{j}{{\left(l+j\right)}^{i+1}} \end{array}$$

(A9c) $$\begin{array}{ccc} & \;\;\;\;\;\;\;\;\;\;\;= & n\psi_{i}\left(l\right)+n\left(\psi_{i}\left(n+l\right)-\psi_{i}\left(l\right)\right)-{\left(-1\right)}^{i}i!\sum_{j=1}^{n-1}\frac{l+j-l}{{\left(l+j\right)}^{i+1}} \end{array}$$

(A9d) $$\begin{array}{ccc} & \;\;\;\;\;\;\;\;\;\;\;\;= & n\psi_{i}\left(n+l\right)-{\left(-1\right)}^{i}i!\sum_{j=1}^{n-1}\frac{1}{{\left(l+j\right)}^{i}}+{\left(-1\right)}^{i}i!l\sum_{j=1}^{n-1}\frac{1}{{\left(l+j\right)}^{i+1}} \end{array}$$

(A9e) $$\begin{array}{ccc} & \;\;\;\;\;\;\;\;\;\;\;\;= & n\psi_{i}\left(n+l\right)+i\left(\psi_{i-1}\left(n+l\right)-\psi_{i-1}\left(l\right)-\frac{{\left(-1\right)}^{i-1}\left(i-1\right)!}{l^{i}}\right) \end{array}$$

(A9f) $$\begin{array}{cc} & \;\;+l\left(\psi_{i}\left(n+l\right)-\psi_{i}\left(l\right)-\frac{{\left(-1\right)}^{i}i!}{l^{i+1}}\right) \end{array}$$

(A9g) $$\begin{array}{ccc} & \;\;\;\;\;\;\;\;\;\;= & \left(n+l\right)\psi_{i}\left(n+l\right)-l\psi_{i}\left(l\right)+i\left(\psi_{i-1}\left(n+l\right)-\psi_{i-1}\left(l\right)\right). \end{array}$$

To prove Equation (A5a), setting l=0 in Equation (A4a) results in

(A10) $$\sum_{k=1}^{n}\psi_{0}\left(k\right)=n\psi_{0}\left(n\right)-n-0\psi_{0}\left(0\right),$$

where the “0×∞” type indeterminacy can be resolved by using the fact that [20]

(A11) $$\psi_{0}\left(\epsilon \right)=-\frac{1}{\epsilon }\left(1-\psi_{0}\left(1\right)\epsilon +o\left(\epsilon^{2}\right)\right),\;\;\;\;\epsilon \to 0,$$

as

(A12) $$0\psi_{0}\left(0\right)=\lim_{\epsilon \to 0}\epsilon \psi_{0}\left(\epsilon \right)=-\lim_{\epsilon \to 0}\left(1-\psi_{0}\left(1\right)\epsilon +o\left(\epsilon^{2}\right)\right)=-1.$$

This proves Equation (A5a). Similarly, to prove Equation (A5b) we set l=0 in Equation (A4b), which gives

(A13) $$\sum_{k=1}^{n}\psi_{i}\left(k\right)=n\psi_{i}\left(n\right)+i\psi_{i-1}\left(n\right)-i\psi_{i-1}\left(0\right)-0\psi_{i}\left(0\right),\;\;\;\;i\geq 1.$$

The indeterminate term $-i\psi_{i-1}\left(0\right)-0\psi_{i}\left(0\right)$ can be handled by the fact that [20]

(A14) $$\psi_{i}\left(\epsilon \right)=\frac{{\left(-1\right)}^{i-1}i!}{\epsilon^{i+1}}+\psi_{i}\left(1\right)+o\left(\epsilon \right),\;\;\;\;\epsilon \to 0,$$

as

(A15a) $$\begin{array}{ccc} i\psi_{i-1}\left(0\right)+0\psi_{i}\left(0\right) & = & \lim_{\epsilon \to 0}\left(i\psi_{i-1}\left(\epsilon \right)+\epsilon \psi_{i}\left(\epsilon \right)\right)\\ & = & \lim_{\epsilon \to 0}\left(\frac{{\left(-1\right)}^{i-2}i!}{\epsilon^{i}}+i\psi_{i-1}\left(1\right)+o\left(\epsilon \right)+\frac{{\left(-1\right)}^{i-1}i!}{\epsilon^{i}}+\epsilon \psi_{i}\left(1\right)+o\left(\epsilon^{2}\right)\right) \end{array}$$

(A15b) $$\begin{array}{ccc} & \;= & \lim_{\epsilon \to 0}\left(i\psi_{i-1}\left(1\right)+o\left(\epsilon \right)+\epsilon \psi_{i}\left(1\right)+o\left(\epsilon^{2}\right)\right)=i\psi_{i-1}\left(1\right), \end{array}$$

which gives Equation (A5b). This completes the proof of Lemma A1.  □

Notice that Equation (A4a) and the special case i=1 of Equation (A4b) also appeared, without proofs, in ([25], Equation (A1)) and ([25], Equation (A7)), respectively.

With the Lemma A1, we turn to the proof of Proposition 1.

**Proof.** In order to utilize the matrix integral approach, we need to parameterize the product channel matrix of Equation (5) as [4]

(A16) $$H=\left(\begin{array}{cc} H & 0_{d_{M}\times \left(d_{0}-d_{M}\right)} \end{array}\right)U,$$

where

(A17) $$H=H_{M}\cdots H_{1}$$

is now the product of *M* square matrices of dimensions $d_{M}\times d_{M}$ and U is a unitary matrix $UU^{†}=I_{d_{M}}$. It follows from Equation (A16) that

(A18) $$HH^{†}=HH^{†},$$

which implies that the product of rectangular matrices H and the product of square matrices H have the same non-zero singular values. The joint density of the square matrices $H_{i}$, i=1,⋯,M, can be written as [4]

(A19) $$\frac{1}{\det{\left(\Sigma \right)}^{d_{M-1}}}\prod_{i=1}^{M}\Gamma_{m}\left(d_{i-1}\right)e^{-\mathrm{tr}\left(\Sigma^{-1}H_{M}H_{M}^{†}\right)}\prod_{i=1}^{M-1}e^{-\mathrm{tr}\left(H_{i}H_{i}^{†}\right)}\prod_{i=1}^{M}\det(H_{i}H_{i}^{†}{)}^{d_{i-1}-m},$$

where the original dimensions are ordered as in Equation (7) with $m=d_{M}$. The above transform is known as the weak commutation relation for products of random matrices [4]. The density (Equation (A19)) is recognized as the product of *M* matrix-variate gamma densities (Equation (A1)), where the parameters $d_{i}$, i=0,⋯,M−1 take the role of the parameter α in Equation (A1). Consequently, the cumulant generating Equation (17) of the random variable Equation (16) can be calculated, in terms of the new matrix-variate density Equation (A19), as

(A20a) $$\begin{array}{ccc} K(s) & = & \ln E\left[e^{s\ln\det\left({\mathrm{HH}}^{†}\right)}\right] \end{array}$$

(A20b) $$\begin{array}{ccc} & \;\;\;= & \ln E\left[\det{\left({\mathrm{HH}}^{†}\right)}^{s}\right] \end{array}$$

(A20c) $$\begin{array}{ccc} & \;\;\;= & \ln E\left[\det{\left({\mathrm{HH}}^{†}\right)}^{s}\right] \end{array}$$

(A20d) $$\begin{array}{ccc} & \;\;\;\;\;= & \ln\prod_{i=1}^{M}E\left[\det{\left(H_{i}H_{i}^{†}\right)}^{s}\right] \end{array}$$

(A20e) $$\begin{array}{cc} \;\;\;\;\;\;\;\;\;\;\;\;\;\;\;\;\;= & \ln\frac{1}{\det{\left(\Sigma \right)}^{d_{M-1}}}\prod_{i=1}^{M}\Gamma_{m}\left(d_{i-1}\right)+\ln\prod_{i=1}^{M-1}\int e^{-\mathrm{tr}\left(H_{i}H_{i}^{†}\right)}\det(H_{i}H_{i}^{†}{)}^{s+d_{i-1}-m}\;dH_{i}H_{i}^{†}+\\ & \ln\int e^{-\mathrm{tr}\left(\Sigma^{-1}H_{M}H_{M}^{†}\right)}\det(H_{M}H_{M}^{†}{)}^{s+d_{M-1}-m}\;dH_{M}H_{M}^{†} \end{array}$$

$$\begin{array}{cc} \;\;\;\;\;\;\;\;\;\;\;\;\;\;\;\;\;= & \ln\frac{1}{\det{\left(\Sigma \right)}^{d_{M-1}}}\prod_{i=1}^{M}\Gamma_{m}\left(d_{i-1}\right)+\ln\prod_{i=1}^{M-1}\Gamma_{m}\left(s+d_{i-1}\right)+\ln\Gamma_{m}\left(s+d_{M-1}\right)\det{\left(\Sigma \right)}^{s+d_{M-1}} \end{array}$$

(A20f) $$\begin{array}{cc} \;\;\;\;\;\;\;\;\;\;\;\;\;\;\;\;\;= & \ln\frac{1}{\det{\left(\Sigma \right)}^{d_{M-1}}}\prod_{i=1}^{M}\Gamma_{m}\left(d_{i-1}\right)+\ln\det{\left(\Sigma \right)}^{s+d_{M-1}}\prod_{i=1}^{M}\Gamma_{m}\left(s+d_{i-1}\right), \end{array}$$

where the second to the last step is established by using the definition of matrix-variate gamma density in Equation (A1). In fact, the results of the integrations can be directly read off from the normalization constant in Equation (A1) with each $d_{i}$ (α in Equation (A1)) replaced by $s+d_{i}$. By the definition of Equation (18), the *i*-th cumulant ${\tilde{\kappa }}_{i}$ of the MIMO Rayleigh product channels Equation (5) can now be computed as

(A21a) $$\begin{array}{ccc} {\tilde{\kappa }}_{i} & = & \frac{\;d^{i}}{\;ds^{i}}K\left(s\right){|}_{s=0} \end{array}$$

(A21b) $$\begin{array}{ccc} & \;\;\;\;\;\;\;\;\;\;= & \frac{\;d^{i}}{\;ds^{i}}\ln\det{\left(\Sigma \right)}^{s}\prod_{j=1}^{M}\Gamma_{m}\left(s+d_{j-1}\right){|}_{s=0} \end{array}$$

(A21c) $$\begin{array}{ccc} & \;\;\;\;\;\;\;\;\;\;\;\;\;\;\;= & \frac{\;d^{i}}{\;ds^{i}}\left(s\ln\det\Sigma +\sum_{j=1}^{M}\sum_{k=0}^{m-1}\ln\Gamma \left(s+d_{j-1}-k\right)\right){|}_{s=0} \end{array}$$

(A21d) $$\begin{array}{ccc} & \;\;\;\;\;\;\;\;\;\;\;= & \delta \left(i-1\right)\ln\det\Sigma +\sum_{j=1}^{M}\sum_{k=0}^{m-1}\psi_{i-1}\left(d_{j-1}-k\right) \end{array}$$

(A21e) $$\begin{array}{ccc} & \;\;\;\;\;\;\;\;\;\;\;\;\;= & \delta \left(i-1\right)\ln\det\Sigma +\sum_{j=1}^{M}\sum_{k=1}^{m}\psi_{i-1}\left(d_{j-1}-m+k\right), \end{array}$$

where we have used the definitions of the multivariate gamma function in Equation (A2) and the polygamma function in Equation (24). By the relation between the cumulants in Equation (19), we now have

(A22a) $$\begin{array}{ccc} \kappa_{1} & = & \sum_{j=1}^{M}\sum_{k=1}^{m}\psi_{0}\left(d_{j-1}-m+k\right)+\ln\det\Sigma +m\ln r\\ & = & m\sum_{j=1}^{M}\psi_{0}\left(d_{j-1}-m\right)+\sum_{j=1}^{M}d_{j-1}\left(\psi_{0}\left(d_{j-1}\right)-\psi_{0}\left(d_{j-1}-m\right)\right)+ \end{array}$$

(A22b) $$\begin{array}{c} m(\ln r-M)+\ln\det\Sigma , \end{array}$$

where we have used Equation (A4a) to arrive at the last equality. This proves Equation (21a). Similarly, by using Equation (A4b) the higher cumulants are simplified as

(A23a) $$\begin{array}{ccc} \kappa_{i} & = & \sum_{j=1}^{M}\sum_{k=1}^{m}\psi_{i-1}\left(d_{j-1}-m+k\right)\\ & = & m\sum_{j=1}^{M}\psi_{i-1}\left(d_{j-1}-m\right)+\sum_{j=1}^{M}d_{j-1}\left(\psi_{i-1}\left(d_{j-1}\right)-\psi_{i-1}\left(d_{j-1}-m\right)\right)+ \end{array}$$

(A23b) $$\begin{array}{c} \left(i-1\right)\sum_{j=1}^{M}\left(\psi_{i-2}\left(d_{j-1}\right)-\psi_{i-2}\left(d_{j-1}-m\right)\right),\;\;\;\;i\geq 2, \end{array}$$

which is Equation (21b). We complete the proof of Proposition 1.  □

We see from Equation (A21) that the use of cumulant generating function turns out to be more convenient than directly studying the moments.

## Appendix B. Proof of Corollary 1

**Proof.** In the case of square channel matrices Equation (27), the starting point to show Equation (28a) is the general expression of Equation (A22a) valid for any channel dimensions $d_{i}$, i=0,⋯,M, which, by using Equation (A5a), is simplified to

(A24a) $$\begin{array}{ccc} \kappa_{1} & = & M\sum_{k=1}^{m}\psi_{0}\left(k\right)+\ln\det\Sigma +m\ln r \end{array}$$

(A24b) $$\begin{array}{ccc} & \;\;\;\;\;= & M\left(m\psi_{0}\left(m\right)-m+1\right)+m\ln r+\ln\det\Sigma . \end{array}$$

Similarly, to show Equation (28b) we start from Equation (A23a), which by using Equation (A5b), is simplified to

(A25a) $$\begin{array}{ccc} \kappa_{i} & = & M\sum_{k=1}^{m}\psi_{i-1}\left(k\right) \end{array}$$

(A25b) $$\begin{array}{ccc} & \;\;\;\;\;\;\;\;\;\;\;\;\;\;\;= & M\left(m\psi_{i-1}\left(m\right)+\left(i-1\right)\left(\psi_{i-2}\left(m\right)-\psi_{i-2}\left(1\right)\right)\right),\;\;\;\;i\geq 2. \end{array}$$

This completes the proof of Corollary 1.  □

## Appendix C. Proof of Proposition 2

**Proof.** As in the proof of Proposition 1, the key here is to utilize the relevant matrix integral instead of using the eigenvalue density Equation (13). In fact, as a truncation of an unitary matrix in Equation (12), the density of the Jacobi MIMO matrix is written as [6]

(A26) $$\frac{\Gamma_{m}\left(l\right)}{\Gamma_{m}\left(n\right)\Gamma_{m}\left(l-n\right)}\det{\left({\mathrm{HH}}^{†}\right)}^{n-m}\det{\left(I_{m}-{\mathrm{HH}}^{†}\right)}^{l-n-m},$$

which is the matrix-variate beta density Equation (A3) with parameters α=n and β=l−n. The corresponding cumulant generating function of Equation (17) over the matrix-variate density of Equation (A26) can now be calculated as

(A27a) $$\begin{array}{ccc} K(s) & = & \ln E\left[e^{s\ln\det\left({\mathrm{HH}}^{†}\right)}\right] \end{array}$$

(A27b) $$\begin{array}{ccc} & \;= & \ln E\left[\det{\left({\mathrm{HH}}^{†}\right)}^{s}\right] \end{array}$$

(A27c) $$\begin{array}{ccc} & \;\;\;\;\;\;\;\;\;\;\;\;\;\;\;\;= & \ln\frac{\Gamma_{m}\left(l\right)}{\Gamma_{m}\left(n\right)\Gamma_{m}\left(l-n\right)}+\ln\int \det{\left({\mathrm{HH}}^{†}\right)}^{s+n-m}\det{\left(I_{m}-{\mathrm{HH}}^{†}\right)}^{l-n-m}\;d{\mathrm{HH}}^{†} \end{array}$$

(A27d) $$\begin{array}{ccc} & \;\;\;\;\;\;\;\;\;\;\;\;= & \ln\frac{\Gamma_{m}\left(l\right)}{\Gamma_{m}\left(n\right)\Gamma_{m}\left(l-n\right)}+\ln\frac{\Gamma_{m}\left(s+n\right)\Gamma_{m}\left(l-n\right)}{\Gamma_{m}\left(s+l\right)}. \end{array}$$

The integration in the second to last line is a trivial deformation of the density of Equation (A26), which was computed by replacing in the normalization constant of Equation (A26) the appearance of α by s+α. By Equation (18), the *i*-th cumulant ${\tilde{\kappa }}_{i}$ of the Jacobi MIMO channels of Equation (12) are now calculated as

(A28a) $$\begin{array}{ccc} {\tilde{\kappa }}_{i} & = & \frac{\;d^{i}}{\;ds^{i}}K\left(s\right){|}_{s=0} \end{array}$$

(A28b) $$\begin{array}{ccc} & \;\;\;\;= & \frac{\;d^{i}}{\;ds^{i}}\ln\frac{\Gamma_{m}\left(s+n\right)}{\Gamma_{m}\left(s+l\right)}{|}_{s=0} \end{array}$$

(A28c) $$\begin{array}{ccc} & \;\;\;\;\;\;\;\;\;\;\;\;\;\;\;= & \frac{\;d^{i}}{\;ds^{i}}\left(\sum_{k=0}^{m-1}\ln\Gamma \left(s+n-k\right)-\sum_{k=0}^{m-1}\ln\Gamma \left(s+l-k\right)\right){|}_{s=0} \end{array}$$

(A28d) $$\begin{array}{ccc} & \;\;\;\;\;\;\;= & \sum_{k=0}^{m-1}\psi_{i-1}\left(n-k\right)-\sum_{k=0}^{m-1}\psi_{i-1}\left(l-k\right) \end{array}$$

(A28e) $$\begin{array}{ccc} & \;\;\;\;\;\;\;\;\;\;= & \sum_{k=1}^{m}\psi_{i-1}\left(n-m+k\right)-\sum_{k=1}^{m}\psi_{i-1}\left(l-m+k\right), \end{array}$$

where the definitions in Equations (24) and (A2) have been utilized. Finally, by using Equation (A4) in Lemma A1 and the relation between the cumulants in Equation (19), we prove Proposition 2.  □
